# Understanding Pain: From Cells to Brain to Individual Perceptions

**DOI:** 10.1080/24740527.2019.1591802

**Published:** 2019-04-02

**Authors:** Karen D. Davis

**Affiliations:** a Division of Brain, Imaging and Behaviour - Systems Neuroscience; Krembil Brain Institute, Toronto, Ontario, Canada; b Department of Surgery, Institute of Medical Science,University of Toronto, Toronto, Ontario, Canada

I was introduced to the field of pain in the late 1970s. At that time, the discovery of an endogenous opiate system fascinated scientists (and budding scientists like me) who asked: why do humans have opiate receptors in their brains and why do our brains manufacture endogenous opiates? Excitement grew as these discoveries led to new ideas about how we experience and potentially alleviate pain. It was soon apparent that the mechanisms underlying our individual experience of pain and the ability to modulate that experience was much more complex than originally conceptualized. This was the cornerstone of my career journey and I have been fortunate to be mentored by and collaborate with some of the seminal pain scientists and clinicians in my career, and to be able to reinvent myself based on technological advances as they emerged. In this talk, I will look back to provide an overview of how this work contributes to understanding pain from the perspective of single cell electrophysiology from primary afferents to the brain in animal models and in humans. I will then discuss the powerful approach of combining brain imaging, psychophysics and behavioural assessments to provide insight into the brain circuitry underlying individual pain experiences. Finally, I will anticipate how these approaches are leading to an understanding of biomarkers that could be developed to predict and guide personalized pain management.

